# Development and Validation of a Multimorbidity Index Predicting Mortality Among Older Chinese Adults

**DOI:** 10.3389/fnagi.2022.767240

**Published:** 2022-03-15

**Authors:** Yan Luo, Ziting Huang, Hui Liu, Huiwen Xu, Hexuan Su, Yuming Chen, Yonghua Hu, Beibei Xu

**Affiliations:** ^1^Department of Epidemiology and Biostatistics, School of Public Health, Peking University, Beijing, China; ^2^Medical Informatics Center, Peking University, Beijing, China

**Keywords:** multimorbidity, measurement, mortality, restrictive association rule mining, older adults

## Abstract

**Objective:**

This study aimed to develop and validate a multimorbidity index using self-reported chronic conditions for predicting 5-year mortality risk.

**Methods:**

We analyzed data from the Chinese Longitudinal Healthy Longevity Survey (CLHLS) and included 11,853 community-dwelling older adults aged 65–84 years. Restrictive association rule mining (ARM) was used to identify disease combinations associated with mortality based on 13 chronic conditions. Data were randomly split into the training (*N* = 8,298) and validation (*N* = 3,555) sets. Two multimorbidity indices with individual diseases only (MI) and disease combinations (MIDC) were developed using hazard ratios (*HR*s) for 5-year morality in the training set. We compared the predictive performance in the validation set between the models using condition count, MI, and MIDC by the concordance (C) statistic, the Integrated Discrimination Improvement (IDI), and the Net Reclassification Index (NRI).

**Results:**

A total of 13 disease combinations were identified. Compared with condition count (C-statistic: 0.710), MIDC (C-statistic: 0.713) showed significantly better discriminative ability (C-statistic: *p* = 0.016; IDI: 0.005, *p* < 0.001; NRI: 0.038, *p* = 0.478). Compared with MI (C-statistic: 0.711), the C-statistic of the model using MIDC was significantly higher (*p* = 0.031), while the IDI was more than 0 but not statistically significant (IDI: 0.003, *p* = 0.090).

**Conclusion:**

Although current multimorbidity status is commonly defined by individual chronic conditions, this study found that the multimorbidity index incorporating disease combinations showed supreme performance in predicting mortality among community-dwelling older adults. These findings suggest a need to consider significant disease combinations when measuring multimorbidity in medical research and clinical practice.

## Introduction

Multimorbidity, commonly defined as the coexistence of multiple chronic diseases and/or conditions within one individual, is prevalent among older populations (Salive, 2013). Multimorbidity has been associated with functional limitations (Kadambi et al., 2020), poor quality of life (Kanesarajah et al., 2018), and mortality (Nunes et al., 2016). With a rapidly aging global population, multimorbidity poses a great economic burden on both individuals and health care systems (Larkin et al., 2021; Soley-Bori et al., 2021). Identifying older patients with multimorbidity at high risks of adverse health outcomes in the community may inform clinicians and public health policymakers to prioritize those groups of people and enable early, effective, and targeted interventions to prevent premature death and reduce health costs (Charlson et al., 2014). Although previous research has made considerable efforts to assess multimorbidity (Nicholson et al., 2019), an international consensus regarding the standard measurement of multimorbidity has yet to be reached (Johnston et al., 2019). Therefore, further explorations for tools to measure multimorbidity are needed for patient care, resource allocation, and the prevention of multimorbidity progression and complications (Wei et al., 2016).

Among all multimorbidity assessments, weighted-multimorbidity indices have been widely used (Stirland et al., 2020). The majority of weighted indices for older adults were developed from hospital patients based on in-patient medical records (Diederichs et al., 2011). Moreover, current multimorbidity indices for community-dwelling adults were mostly developed from young or middle-aged populations in western countries (Lee et al., 2006; Wister et al., 2015; Wei et al., 2016). Given disparities in the spectrum of diseases in different regions and populations worldwide, these indices might not be generalizable to older Chinese adults (GBD 2017 Causes of Death Collaborators, 2018; Zhou et al., 2019).

Although current multimorbidity indices are able to distinguish and depict the influences of chronic conditions on mortality, little research considers the interaction of those conditions in the indices. Previous studies showed that disease combinations had discordant effects on mortality, but most of them identified disease combinations through traditional methods (Caughey et al., 2010; Ferrer et al., 2017). Association rule mining (ARM) is a data-driven approach that has been applied previously to discover significant disease combinations (Held et al., 2016; Yao et al., 2020). Therefore, a multimorbidity index considering the effects of important disease combinations derived from ARM might better capture the whole impact of multiple chronic conditions on mortality.

Developing an explicit and validated measurement tool is significant to help assess the health risks based on diagnoses of chronic diseases and classify older adults into different risk groups for targeted clinical treatment and health management. Moreover, identifying important combinations of diseases may facilitate the evidence-based co-treatment and further investigation into underlying mechanisms (Brown and Thorsteinsson, 2020). Therefore, this study aimed to investigate disease combinations significantly associated with 5-year mortality among community-dwelling older Chinese adults aged 65–84 years from the Chinese Longitudinal Healthy Longevity Survey (CLHLS), as well as develop a multimorbidity index incorporating disease combinations to predict 5-year mortality.

## Materials and Methods

### Participants

This study used data from six waves of the CLHLS 2000–2014. The CLHLS is a prospective longitudinal study with the aim to assess determinants of healthy longevity in China. The survey was first conducted in 1998 and subsequent surveys were carried out every 2 or 3 years. The surveys were conducted in half of the counties and cities randomly selected from 23 provinces in China. The participants were enrolled *via* multistage disproportionate sampling (Gu, 2008). More details about the study design were provided elsewhere (Zeng et al., 2017). Duke University Medical Health System’s Institutional Review Board (IRB), the National Bureau of Statistics of China, and the Ethical Committee of Social Science Division of Peking University reviewed and approved ethics for CLHLS. Written informed consent was obtained from participants or their proxies.

Among 40,359 participants from the six waves, we only included participants aged 65–84 years at baseline (*N* = 14,148), who were frequently defined as “older adults” in previous research and accounted for approximately 94% of adults aged ≥65 years in China (United Nations [UN], 2019; Greer et al., 2021). Additionally, the median survival time of participants aged 65–69, 70–74, 75–79, and 80–84 years at baseline was 16.9, 12.0, 8.8, and 5.8 years, respectively, all of which were more than 5 years (Supplementary Figure 1). After excluding those who were lost to follow up after baseline survey (*N* = 2,295), a total of 11,853 participants, with the mean follow-up of 4.1 [standard deviation (SD): 1.4] years, were included for analyses. The flowchart of the participant selection and sample sizes of the dynamic cohort is shown in Supplementary Figure 2 and Supplementary Table 1.

### Chronic Conditions

In this study, 13 chronic diseases or conditions (abbreviated hereafter as chronic conditions) at baseline were included, covering most somatic diseases and mental disorders frequently used in measuring multimorbidity (Diederichs et al., 2011; The Academy of Medical Sciences, 2018; Stirland et al., 2020). In addition, seven chronic conditions were ascertained by asking participants whether a doctor told them that they had diabetes, cerebrovascular disease, heart disease, cancer, lung disease (bronchitis, emphysema, asthma, pneumonia, and tuberculosis), Parkinson’s disease, and arthritis. Blood pressure was measured by a trained physician with the electronic sphygmomanometer (Omron HEM-7200 Monitor) and the mean of two repeated measures was calculated. Participants were considered hypertensive, if their systolic blood pressure was ≥140 mmHg and/or diastolic blood pressure ≥90 mmHg, or if they self-reported being diagnosed with hypertension by the physicians (Writing Group of 2018 Chinese Guidelines for the Management of Hypertension et al., 2019). Cognitive impairment was defined as having either self-reported dementia or impaired cognitive function. Cognitive function was measured using the Chinese version of the Mini-Mental State Examination (MMSE). The MMSE score ranged from 0 to 30, and impaired cognitive function was defined as an MMSE score ≤18 as previously validated (Zhang et al., 2010). Depressive symptoms were assessed by a five-item Likert scale with scores ranging from 0 to 20 and the acceptable internal consistency reliability (Cronbach’s α = 0.66) (Shen et al., 2019). This scale has been commonly used to identify depressive symptoms in several studies using CLHLS data (Yi and Vaupel, 2004; Zeng et al., 2013; Feng et al., 2015; Shen et al., 2019). Those with the depression score ≤7, which was the median of all participants, were considered to have depressive symptoms. Participants were identified as having vision impairment, if they were unable to distinguish the break in the circle or see the circle clearly or blind, and/or reported having cataracts and/or glaucoma (Cao et al., 2021). Participants were considered to have hearing impairment, if they cannot hear clearly what the interviewers said despite using a hearing aid or cannot hear anything (Zhang et al., 2020). Sensory impairment was defined as having hearing impairment and/or vision impairment. Bedridden status was defined from either self-reported bedsore or being permanently bedridden in the past 2 years (Mervis and Phillips, 2019). Tooth loss was defined as having no natural teeth and without dentures (Yuan et al., 2020). Definitions of chronic conditions are shown in Supplementary Table 2. Participants answered questions about cognitive function and depressive symptoms on their own, while proxy respondents would answer other questions, if participants were unable to complete the interview due to cognitive and linguistic impairments (Gu, 2008; Lv et al., 2019). All diseases and conditions were defined as the binary variables.

### Mortality

All-cause mortality was ascertained through a face-to-face interview with a close family member for those interviewees who had died during the follow-ups (Zeng et al., 2008). All-cause mortality is a common choice for an adverse outcome to understand the progression and severity of an exposure (e.g., chronic diseases), with a minor bias but easy to measure (Ferguson et al., 2013; Weiss, 2014). Additionally, mortality is one of the most commonly used outcomes to develop the multimorbidity index, which could facilitate the comparison between our indices and established indices (Nicholson et al., 2019). Follow-up time was defined as the period from the date of the baseline visit to the date of death or the last follow-up. At the 5-year follow-up, survivors were censored, which is the standard cutoff for evaluating the effect of screening or treatment for older adults with chronic diseases, such as cancer (Miller et al., 2020). Participants who were lost to follow-up during the 5-year follow-up were censored at the time of the last survey. The proportions for participants who were lost to follow-up or died within the 5-year follow-up were 16.3 and 25.2%, respectively.

### Statistical Analyses

Baseline characteristics were summarized using frequencies (percentages) for categorical variables and median [interquartile range (IQR)] for continuous variables. The chi-square tests for categorical variables and Mann–Whitney *U*-test for continuous variables were used to compare baseline characteristics between survivors and non-survivors at 5-year follow-up (Supplementary Table 3).

Association rule mining was performed to identify the pairs of chronic conditions associated with mortality. ARM allows the identification of novel and potentially relevant associations of diseases without stating *a priori* hypotheses (Prados-Torres et al., 2014; Held et al., 2016). For association rules like ({A}→{B}) with an “antecedent” {A} and a “consequent” {B}, “support” refers to the frequency of the particular combination of A and B; “confidence” refers to how frequently B occurs conditionally on A; “lift” refers to how much more frequently A and B occur together compared with how often would be expected under statistical independence (Yao et al., 2020). The parameters of ARM were set as follows: minimum support >1.5%, minimum confidence >10%, lift >1.0, the number of items in the antecedent was limited to 2, and the consequent was restricted to 5-year mortality. Disease combinations were ascertained as the antecedents of association rules matching all parameters. For example, a disease combination of hypertension and sensory impairment was defined as the coexistence of these two chronic conditions based upon the rule of ({Hypertension, Sensory impairment}→{5-year mortality}).

Participants aged 65–84 years at baseline were randomly divided into training (70% of analytic sample) and validation (30% of analytic sample) sets. Multimorbidity indices with individual diseases (MI) or disease combinations (MIDC) were developed by Cox proportional hazards models in the training set. Model 1 included age, sex, and chronic conditions as independent variables, and Model 2 further added disease combinations derived from the restrictive ARM. All independent variables were based on baseline information, without considering disease evolution over time. The outcome was 5-year mortality for both Model 1 and Model 2. Adjusted hazard ratios (*HR*s) estimated by Model 1 were used to assign the weights for conditions in MI, while HRs from Model 2 were used for conditions and disease combinations in MIDC. Consistent with previous studies, a condition or disease combination with an *HR* = 1.00–1.19, 1.20–1.49, and ≥1.50, was assigned a weight of 1, 2, and 3, respectively (Charlson et al., 1987; Mukherjee et al., 2011). MI and MIDC were calculated by summing the weighted scores of conditions and/or disease combinations. The conditions or disease combinations with *HR* < 1 were excluded for the final calculation of MI and MIDC. More details of the process to develop MI and MIDC are shown in Supplementary Figure 3.

The base model with age and sex, and three models added simple condition count, MI, and MIDC at baseline, respectively, were employed to predict 5-year mortality in the validation set. We compared the performance to predict 5-year mortality between every two of the models above by the concordance (C) statistic, the Integrated Discrimination Improvement (IDI), and the continuous Net Reclassification Index (NRI). The C-statistic generally ranging from 0.5 to 1.0 is an overall measure to compare the discrimination power of risk prediction models. A C-statistic closer to one indicates better performance of the predictive models (Harrell et al., 1996; Schroder et al., 2011). The IDI is defined as the comparative improvement of a new model in sensitivity and specificity for events. The NRI assesses the increase of model-based probabilities for events and the decrease of the probabilities for non-events. A positive IDI or NRI indicates that the new model predicts better than the comparator model (Kerr et al., 2011; Uno et al., 2013).

All data management and analyses were performed by R software version 4.0.0 (R Foundation for Statistical Computing, Vienna, Austria). Two-tailed *p* < 0.05 was considered statistically significant.

## Results

Baseline characteristics of all participants are summarized in Table 1. Among 11,853 participants, the median age was 76.0 (IQR: 69.0, 81.0) years and 53.0% were men. Hypertension, depressive symptom, and sensory impairment were the most common conditions, with the prevalence rates of 55.6, 36.0, and 23.7%, respectively.

**TABLE 1:** The baseline characteristics of training and validation sets*.

Characteristics	Total	Training set	Validation set	*P*-value^†^
	(*N* = 11,853)	(*N* = 8,298)	(*N* = 3,555)	
Age (years), median (IQR)	76.0 (69.0, 81.0)	76.0 (69.0, 81.0)	76.0 (69.0, 81.0)	0.434
Male	6,287 (53.0)	4,398 (53.0)	1,889 (53.1)	0.908
Hypertension	6,596 (55.6)	4,626 (55.7)	1,970 (55.4)	0.753
Diabetes	384 (3.2)	278 (3.4)	106 (3.0)	0.326
Heart disease	1,240 (10.5)	893 (10.8)	347 (9.8)	0.110
Cerebrovascular disease	747 (6.3)	517 (6.2)	230 (6.5)	0.653
Parkinson’s disease	58 (0.5)	43 (0.5)	15 (0.4)	0.586
Arthritis	2,165 (18.3)	1,514 (18.2)	651 (18.3)	0.952
Tooth loss	712 (6.0)	501 (6.0)	211 (5.9)	0.863
Lung disease	1,585 (13.4)	1,084 (13.1)	501 (14.1)	0.139
Cancer	61 (0.5)	47 (0.6)	14 (0.4)	0.288
Sensory impairment	2,815 (23.7)	1,991 (24.0)	824 (23.2)	0.351
Cognitive impairment	568 (4.8)	392 (4.7)	176 (5.0)	0.629
Bedridden status	132 (1.1)	90 (1.1)	42 (1.2)	0.715
Depressive symptoms	4,265 (36.0)	3,011 (36.3)	1,254 (35.3)	0.303

*IQR, interquartile range.*

*Data are presented as n (%) unless otherwise indicated.

*^†^The value of p was calculated using chi-square tests for categorical variables and Mann–Whitney U-test for continuous variables.*

Table 2 presents the results of restrictive ARM. Among all the participants, a total of 13 disease combinations were identified. The disease combination with the highest lift was hypertension and cognitive impairment. Older patients with hypertension and depressive symptoms, who died during a 5-year follow-up accounted for 6.6% among all participants. The prevalence rates of the co-occurrence of hypertension and depressive symptoms were 20.4% among all participants and 32.6% among participants who died during a 5-year follow-up.

**TABLE 2:** Results of association rule mining among participants aged 65–84 years (*N* = 11,853).

Rules		Support (%)*	Confidence (%)^†^	Lift	Prevalence (%)^††^
Antecedent	Consequent				
Hypertension, depressive symptoms	Five-year mortality	6.6	32.6	1.3	20.4
Hypertension, sensory impairment		5.1	36.0	1.4	14.2
Sensory impairment, depressive symptoms		4.6	39.2	1.6	11.6
Hypertension, lung disease		2.5	33.9	1.3	7.5
Lung disease, depressive symptoms		2.2	39.2	1.6	5.7
Arthritis, depressive symptoms		2.0	25.9	1.0	7.9
Cognitive impairment, depressive symptoms		2.0	58.0	2.3	3.4
Hypertension, heart disease		2.0	26.2	1.0	7.5
Sensory impairment, cognitive impairment		1.9	56.7	2.3	3.4
Hypertension, cerebrovascular disease		1.7	37.2	1.5	4.6
Hypertension, cognitive impairment		1.7	58.9	2.3	2.8
Hypertension, tooth loss		1.6	45.5	1.8	3.5
Lung disease, sensory impairment		1.6	40.6	1.6	3.9

*Support was the proportion of patients with the disease combination and died during a 5-year follow-up among all participants.

*^†^Confidence was the proportion of patients with the disease combination among participants who died during a 5-year follow-up.*

*^††^Prevalence was the proportion of patients with the disease combination among all participants.*

**FIGURE 1:**
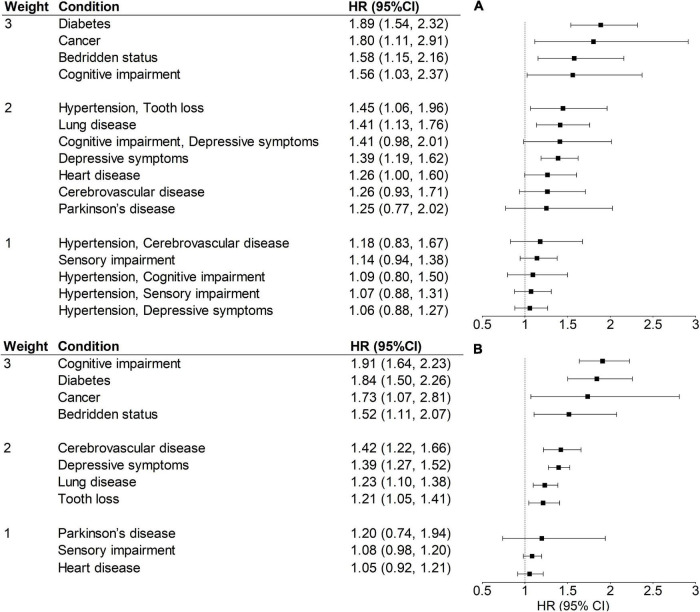
Weights, *HR*s, and 95% CIs of MIDC **(A)** and MI **(B)** for 5-year mortality in the training set (*N* = 8,298). All models were adjusted for age and sex. A condition or disease combination with an *HR* = 1.00–1.19, 1.20–1.49, and ≥1.50, was assigned a weight of 1, 2, and 3, respectively. HR, hazard ratio; CI, confidence interval; MI, multimorbidity index with individual diseases; MIDC, multimorbidity index incorporating disease combinations.

Figure 1 shows the *HR*s and weights of each condition or disease combination. Cognitive impairment, diabetes, cancer, and bedridden status were the conditions with a weight of 3, while hypertension and arthritis with *HR* < 1 were excluded for the calculation of both MI and MIDC. Each of the six disease combinations derived from restrictive ARM and with *HR* > 1 was assigned a weight of 1 or 2. The total ranges of MI and MIDC were 0–23 and 0–31, respectively. A higher score indicates a greater burden of multimorbidity. The distributions of these two indices in the validation set were shown in Supplementary Figure 4. The Cronbach’s α coefficient of MIDC in the validation set was 0.65, indicating acceptable internal consistency reliability. For validity, Pearson’s correlation coefficients (Supplementary Table 4) showed that the MIDC was significantly related to condition count (*r* = 0.88, *p* < 0.001) and MI (*r* = 0.95, *p* < 0.001).

The results of predictive validity analyses are presented in Table 3. The C-statistics of condition count, MI, and MIDC were significantly higher for mortality prediction than that of the base model with only age and sex (all *p* < 0.001). Moreover, the reclassification measures of discrimination showed that all models, such as condition count, MI, and MIDC performed better in predicting 5-year mortality than the base model (IDI: more than 0, all *p* < 0.001; NRI: more than 0, all *p* < 0.001). Compared with condition count, MIDC showed significantly better discriminative ability (C-statistic: *p* = 0.016; IDI: 0.005, *p* < 0.001; NRI: 0.038, *p* = 0.478). Compared with MI, the C-statistic of the model using MIDC was significantly higher (*p* = 0.031), while the IDI was more than 0 but not statistically significant (IDI: 0.003, *p* = 0.090).

**TABLE 3:** C-statistics, IDIs, and NRIs for 5-year mortality in the validation set (*N* = 3,555).

Measures of multimorbidity	*C*-statistic	*P*-value	IDI	*P*-value	NRI	*P*-value
Base model*	0.701	Reference	–	Reference	–	Reference
Base model + *Condition count*	0.710	<0.001	0.017	<0.001	0.083	<0.001
Base model + *MI*	0.711	<0.001	0.020	<0.001	0.101	<0.001
Base model + *MIDC*	0.713	<0.001	0.022	<0.001	0.110	<0.001
Base model + *Condition count*	0.710	Reference	–	Reference	–	Reference
Base model + *MI*	0.711	0.231	0.002	0.259	0.017	0.478
Base model + *MIDC*	0.713	0.016	0.005	<0.001	0.038	0.478
Base model + *MI*	0.711	Reference	–	Reference	–	Reference
Base model + *MIDC*	0.713	0.031	0.003	0.090	−0.019	0.965

*IDI, Integrated Discrimination Improvement; NRI, Net Reclassification Index; MI, multimorbidity index with individual diseases; MIDC, multimorbidity index incorporating disease combinations.*

*The base model included age and sex as independent variables.

## Discussion

To the best of our knowledge, this is one of the first studies to develop and validate the MIDC to predict 5-year mortality in community-dwelling older Chinese adults aged 65–84 years. The MIDC showed greater predictive performance than simple condition count and MI in predicting mortality. This suggests that it is of great importance to consider the effect of specific disease combinations when assessing the impact of coexisting chronic conditions on mortality in older adults.

In our study, we found that 13 disease combinations and 5-year mortality were co-occurring more frequently than expected in older adults. Among these combinations, dyads of *hypertension and depressive symptoms*, as well as *hypertension and sensory impairment*, were most prevalent, which was in line with a large body of literature and could be explained by underlying pathophysiological mechanisms (de Moraes Marchiori et al., 2006; Bhargava et al., 2012; Maatouk et al., 2016; Crews et al., 2017; Jin et al., 2019). It has been reported that the co-occurrence between hypertension and depressive symptoms could be because of neurobiological changes caused by vascular diseases and psychosocial stressors due to the diagnosis of hypertension (Maatouk et al., 2016; Jin et al., 2019). Microvascular damage in the retina and cochlea associated with elevated blood pressure, such as vascular stenosis and hemorrhage, has been proposed as a potential mechanism of hearing impairment and vision loss (de Moraes Marchiori et al., 2006; Bhargava et al., 2012). These findings highlighted that somatic diseases associated with comorbid mental health problems and age-related sensory changes in older people required more attention in primary care and clinical settings.

According to the cox regression results, higher mortality risks of the disease pairs, such as *hypertension and tooth loss*, and *cognitive impairment and depressive symptoms*, were observed. There has been little research comparing the effect of coexistence of hypertension and tooth loss on mortality to that of other disease pairs. However, a previous study reported that tooth loss increased the risk of hypertension due to insufficient intake of fiber and chronic systemic inflammation caused by periodontal disease (Da et al., 2019). Another cohort study of 7,674 Sweden adults also showed that tooth loss was significantly associated with all-cause mortality and cardiovascular diseases mortality (Holmlund et al., 2010). Furthermore, periodontitis has been shown to significantly increase the magnitude of endothelial dysfunction in patients with hypertension, which may accelerate the progression of carotid atherosclerosis and incidence of stroke, myocardial infarction, and cardiovascular diseases death (Higashi et al., 2008; Desvarieux et al., 2010). These results may imply that older people with both hypertension and tooth loss may be more likely to have cardiovascular diseases and further lead to premature mortality. Moreover, previous research has revealed that older patients with dementia and depression had higher mortality risks than those with most of the other chronic conditions (Schafer et al., 2018; Zheng et al., 2021). Late-life depression could interact with cognitive impairment by sharing common underlying pathogenetic mechanisms related to ischemic brain lesions (Linnemann and Lang, 2020). The co-occurrence of cognitive impairment and depression may indicate severe vascular dysfunction, leading to a high cardiovascular mortality risk (Georgakis et al., 2016). As a result, the combinations of these diseases may provide additional valuable information on predicting mortality in older patients with multimorbidity.

Our study compared the performance of condition count, MI, and MIDC in predicting mortality. As expected, based on C-statistics, we found that multimorbidity, measured by condition count, MI, and MIDC, showed significantly better discriminative ability in predicting mortality than age and sex. Although age has been found to be a strong predictor of mortality in several multimorbidity indices, multimorbidity should be additionally considered when assessing the mortality risks of older adults, as previously validated (Charlson et al., 1994; Lee et al., 2006; Nunes et al., 2016). Furthermore, in accordance with the study conducted in older adults aged ≥65 years in Canada, our finding showed that condition count, which was easy to use and understand in clinical settings, has an acceptable prediction performance for mortality among older adults (Quail et al., 2011).

In this study, MIDC performed better than condition count in predicting 5-year mortality. As numerous studies have noted that the association between different chronic conditions and mortality varies among older adults, considering the types and severity of diseases might capture the heterogeneity of their impacts on health (Wei et al., 2016; Stanley and Sarfati, 2017). In addition, we also found that the C-statistic of MIDC was significantly higher than that of MI. Prior research has indicated that specific combinations of diseases, especially cardiovascular and neuropsychiatric disease patterns, may have a synergistic effect on disability or mortality, which partially supports our findings of better performance of MIDC (Jackson et al., 2015; Zheng et al., 2021). Therefore, measuring multimorbidity through multimorbidity index including the effects of specific disease combinations provides a more nuanced risk classification of older patients with multimorbidity and a qualitative dimension that can be useful in clinical practice and research (Johnston et al., 2019; Stirland et al., 2020).

One of the strengths of this study is that we developed a multimorbidity index for community-dwelling older Chinese adults using the data from a multi-province longitudinal study. This approach can support that our index has good generalizability among this population. Moreover, our study included 13 chronic conditions covering most of the conditions prevalent among older Chinese adults and widely used in previous multimorbidity indices (Diederichs et al., 2011; Wang et al., 2020). In addition, compared to previous multimorbidity indices using only individual diseases, we conducted the restrictive ARM to obtain disease combinations important to predict mortality and considered the joint effects of multiple chronic diseases in our index. However, our study has several limitations. First, since our index focused on community-dwelling older adults, it is likely that our index will not be applicable to a nursing home and other institutional populations. Second, most of the chronic conditions were assessed by self-reported questionnaires rather than clinical records, which might lead to recall bias, especially diabetes. However, previous studies also emphasized the importance of self-reported information on a population level since the accuracy of self-reported diseases may reflect the participants’ health literacy (e.g., the awareness of diseases), which could improve the generalization of our index in the community (Smith et al., 2008; van den Akker et al., 2015). Third, the MMSE is not a sensitive measurement of cognitive impairment, and future studies need to assess cognitive function by better tools such as the Montreal Cognitive Assessment (MoCA) (Nasreddine et al., 2005; Arevalo-Rodriguez et al., 2015). Last, we did not have information on the severity, and treatment of each condition, and their influence on mortality needs to be considered in further research.

## Conclusion

Multimorbidity index incorporating disease combinations, followed by multimorbidity index with individual diseases, improved the accuracy of 5-year mortality prediction. This may provide a tool for overall risk stratification, care management, and healthcare resource allocation among community-dwelling older Chinese adults. Moreover, our findings may also imply to researchers that considering significant disease combinations to capture synergistic effects is extremely valuable in predicting mortality among older adults with multimorbidity. Further studies are needed to investigate the association of the MIDC with other health outcomes and validate the MIDC in other populations. In addition, condition count may also be a good choice for measuring multimorbidity for its simplicity and the ease of data ascertainment.

## Data Availability Statement

Publicly available datasets were analyzed in this study. This data can be found here: Peking University Open Research Data Platform, https://opendata.pku.edu.cn/dataset.xhtml?persistentId=doi: 10.18170/DVN/WBO7LK.

## Ethics Statement

The studies involving human participants were reviewed and approved by the Duke University Medical Health System’s Institutional Review Board (IRB), the National Bureau of Statistics of China, and the Ethical Committee of Social Science Division of Peking University. The patients/participants provided their written informed consent to participate in this study.

## Author Contributions

YL, HL, and BX designed the study. YL acquired the data, performed the statistical analyses, and drafted the manuscript. YL, ZH, HL, HX, HS, YC, YH, and BX revised the manuscript. All authors approved the final version of the manuscript.

## Conflict of Interest

The authors declare that the research was conducted in the absence of any commercial or financial relationships that could be construed as a potential conflict of interest.

## Publisher’s Note

All claims expressed in this article are solely those of the authors and do not necessarily represent those of their affiliated organizations, or those of the publisher, the editors and the reviewers. Any product that may be evaluated in this article, or claim that may be made by its manufacturer, is not guaranteed or endorsed by the publisher.
