# Supplementary material for: Development and Validation of a Multimorbidity Index Predicting Mortality Among Older Chinese Adults
Source: Front Aging Neurosci. 2022 Mar 15;14:767240. doi: 10.3389/fnagi.2022.767240 (PMC8965437; doi:10.3389/fnagi.2022.767240)
Supplement: Supplementary file 1 [file Table_1.docx]

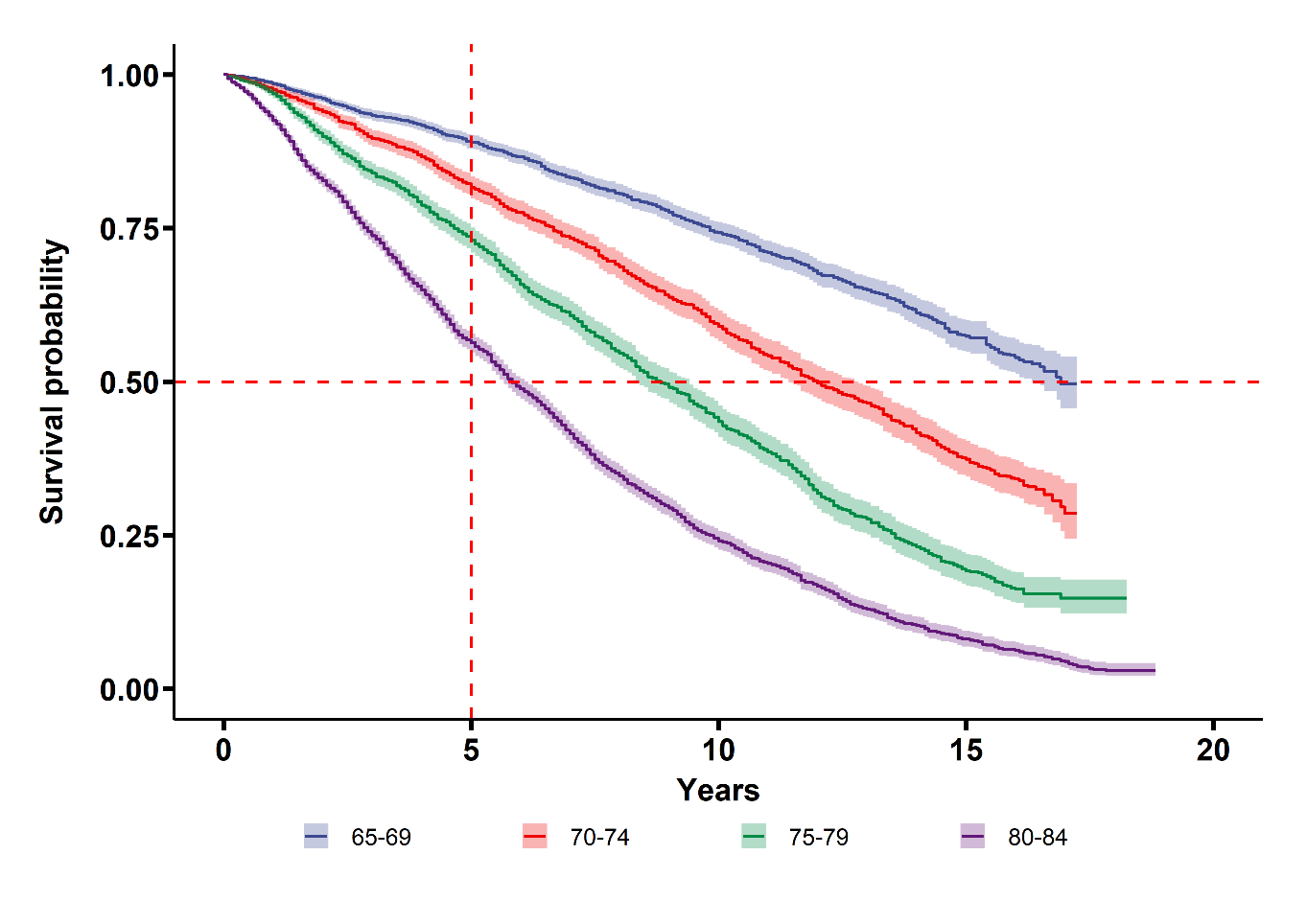


**Supplementary Figure 1.** Survival curves by age at baseline among participants aged 65–84 years (N=11,853).


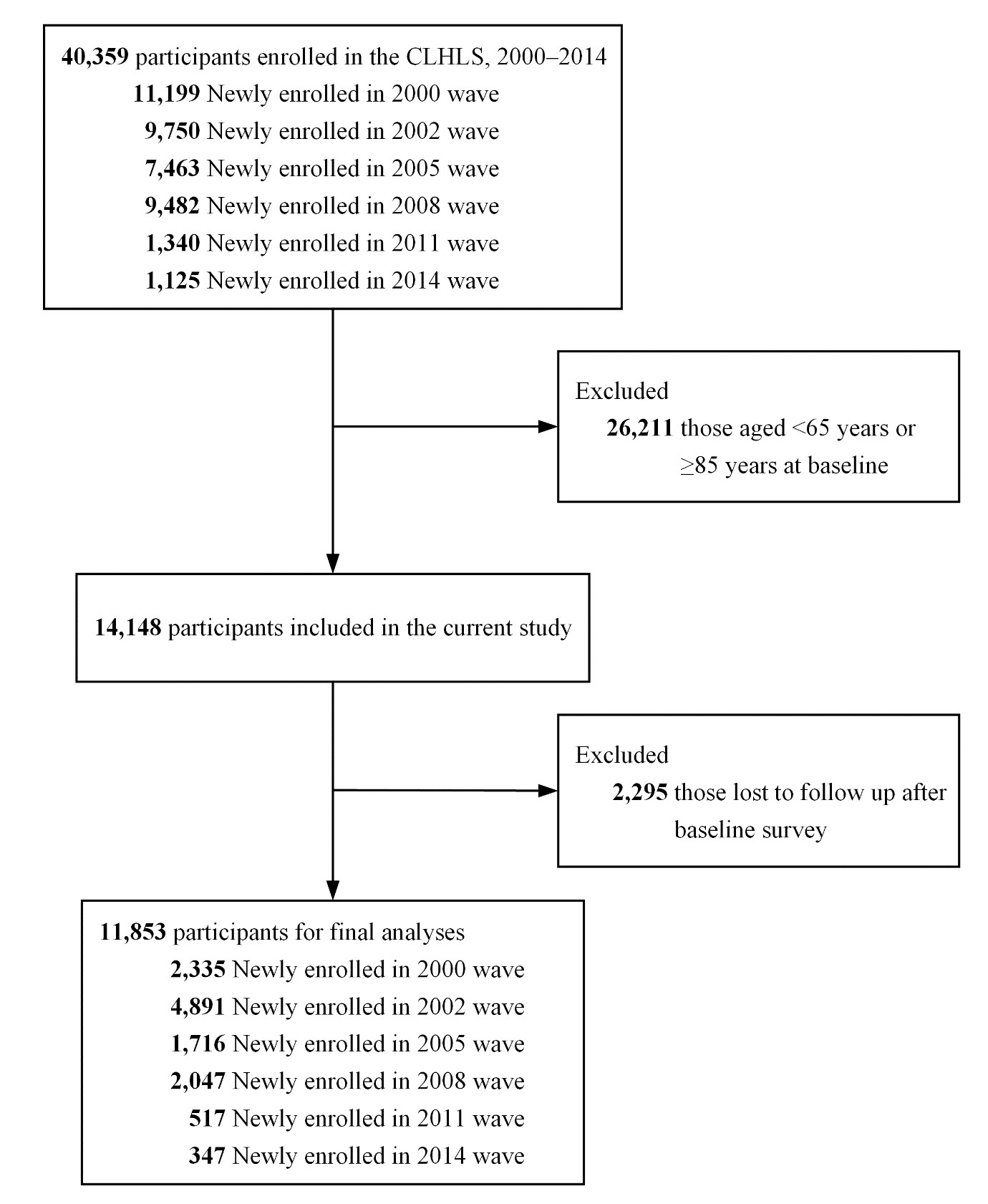


**Supplementary Figure 2.** Flowchart of participants selection. CLHLS, Chinese Longitudinal Healthy Longevity Survey

**Supplementary Table 1.** Sample sizes of the dynamic cohort

| **Baseline**  **survey** | **Follow-up survey** | | | | | |
| --- | --- | --- | --- | --- | --- | --- |
|  | 2002 | 2005 | 2008 | 2011 | 2014 | 2018 |
| 2000  (N=2,335) | Died 444  Lost to follow-up 0  Survived 1,891 | Died 556  Lost to follow-up 235  Survived 1,100 | Died 383  Lost to follow-up 206  Survived 511 | Died 209  Lost to follow-up 63  Survived 239 | Died 119  Lost to follow-up 10  Survived 110 | Died 49  Lost to follow-up 39  Survived 22 |
| 2002  (N=4,891) |  | Died 699  Lost to follow-up 0  Survived 4,192 | Died 623  Lost to follow-up 722  Survived 2,847 | Died 521  Lost to follow-up 317  Survived 2,009 | Died 437  Lost to follow-up 97  Survived 1,475 | Died 384  Lost to follow-up 352  Survived 739 |
| 2005  (N=1,716) |  |  | Died 189  Lost to follow-up 0  Survived 1,527 | Died 178  Lost to follow-up 248  Survived 1,101 | Died 140  Loss to follow-up 74  Survived 887 | Died 159  Lost to follow-up 221  Survived 507 |
| 2008  (N=2,047) |  |  |  | Died 277  Lost to follow-up 0  Survived 1,770 | Died 208  Lost to follow-up 176  Survived 1,386 | Died 261  Lost to follow-up 292  Survived 833 |
| 2011  (N=517) |  |  |  |  | Died 29  Lost to follow-up  Survived 488 | Died 71  Lost to follow-up 55  Survived 362 |
| 2014  (N=347) |  |  |  |  |  | Died 62  Loss to follow-up 0  Survived 285 |

**Supplementary Table 2.** Definition of each chronic condition included

| Chronic conditions | Definition |
| --- | --- |
| Diabetes | Self-reported diabetes |
| Heart disease | Self-reported heart diseases |
| Cerebrovascular disease | Self-reported cerebrovascular disease |
| Cancer | Self-reported cancer |
| Lung disease | Self-reported lung disease (including bronchitis, emphysema, asthma, pneumonia, and tuberculosis) |
| Parkinson’s disease | Self-reported Parkinson’s disease |
| Arthritis | Self-reported arthritis |
| Hypertension | Self-reported hypertension  or  Measured systolic blood pressure/diastolic blood pressure ≥140/90 mmHg |
| Cognitive impairment | Self-reported dementia  or  MMSE score ≤18 |
| Depressive symptoms | A five-item Likert scale ≤7 |
| Sensory impairment | Self-reported cataracts and/or glaucoma  or  Unable to distinguish the break in the circle/unable to see the circle clearly/being blind (All participants are needed to complete the visual function assessment)  or  Unable to hear clearly what the interviewers said despite using a hearing aid/unable to hear anything (Participants are identified as having no hearing impairment if they can hear what the interviewers said whether they use the hearing aid) |
| Bedridden status | Self-reported bedsore  or  Permanently bedridden in the past two years |
| Tooth loss | Have no natural teeth  and  Do not use the dentures |

MMSE, Mini-Mental State Examination.

**Supplementary Table 3.** Baseline characteristics of survivors and non-survivors at 5-year follow-up^*^

| Characteristics | Overall | | Survivors | | Non-survivors | | *P*-value^†^ |
| --- | --- | --- | --- | --- | --- | --- | --- |
|  | (N=11,853) | | (N=8,870) | | (N=2,983) | |  |
| Age (years), median (IQR) | 76.0 | (69.0, 81.0) | 74.0 | (67.0, 80.0) | 80.0 | (75.0, 82.0) | <0.001 |
| Male | 6,287 | (53.0) | 4,563 | (51.4) | 1,724 | (57.8) | <0.001 |
| Hypertension | 6,596 | (55.6) | 4,843 | (54.6) | 1,753 | (58.8) | <0.001 |
| Diabetes | 384 | (3.2) | 257 | (2.9) | 127 | (4.3) | <0.001 |
| Heart disease | 1,240 | (10.5) | 902 | (10.2) | 338 | (11.3) | 0.079 |
| Cerebrovascular disease | 747 | (6.3) | 477 | (5.4) | 270 | (9.1) | <0.001 |
| Parkinson’s disease | 58 | (0.5) | 33 | (0.4) | 25 | (0.8) | 0.003 |
| Arthritis | 2,165 | (18.3) | 1,664 | (18.8) | 501 | (16.8) | 0.018 |
| Tooth loss | 712 | (6.0) | 420 | (4.7) | 292 | (9.8) | <0.001 |
| Lung disease | 1,585 | (13.4) | 1,062 | (12.0) | 523 | (17.5) | <0.001 |
| Cancer | 61 | (0.5) | 35 | (0.4) | 26 | (0.9) | 0.003 |
| Sensory impairment | 2,815 | (23.7) | 1,863 | (21.0) | 952 | (31.9) | <0.001 |
| Cognitive impairment | 568 | (4.8) | 258 | (2.9) | 310 | (10.4) | <0.001 |
| Bedridden status | 132 | (1.1) | 70 | (0.8) | 62 | (2.1) | <0.001 |
| Depressive symptoms | 4,265 | (36.0) | 2,945 | (33.2) | 1,320 | (44.3) | <0.001 |

IQR, interquartile range.

^*^Data are presented as n (%) unless otherwise indicated.

^†^*P* value was calculated using Chi-square tests for categorical variables and Mann-Whitney U test for continuous variables.


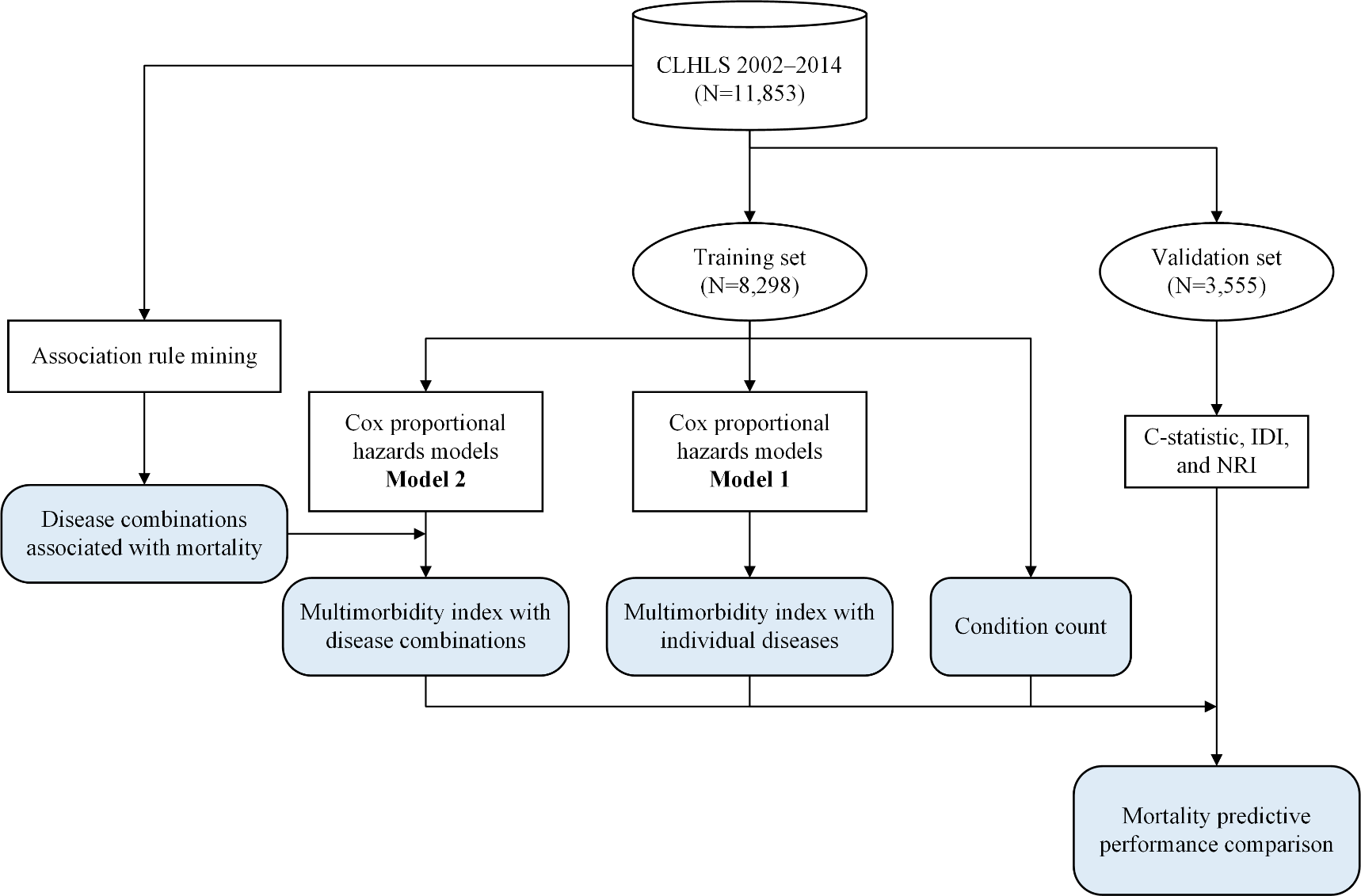


**Supplementary Figure 3.** Flow diagram of the development of multimorbidity index with individual diseases or disease combinations. Model 1 included age, sex, and chronic conditions; Model 2 included age, sex, chronic conditions, and disease combinations derived from the restrictive Association rule mining. CLHLS, Chinese Longitudinal Healthy Longevity Survey; IDI, Integrated Discrimination Improvement; NRI, Net Reclassification Index





**Supplementary Figure 4.** Distributions of condition count, MI, and MIDC in the validation set. MI, multimorbidity index with individual diseases; MIDC, multimorbidity index incorporating disease combinations.

**Supplementary Table 4.** Pearson’s correlation coefficients between Condition count, MI, and MIDC in the validation set (N=3,555)

| Measures of multimorbidity | Condition count | MI | MIDC |
| --- | --- | --- | --- |
| Condition count | 1.00 | 0.85^***^ | 0.88^***^ |
| MI | 0.85^***^ | 1.00 | 0.95^***^ |
| MIDC | 0.88^***^ | 0.95^***^ | 1.00 |

^*^*P*<0.05, ^**^*P*<0.01, ^***^*P*<0.001.

MI, multimorbidity index with individual diseases; MIDC, multimorbidity index incorporating disease combinations.
